# First results of a national deployment of a fully automated central-line-associated bloodstream infection (CLABSI) surveillance system, Switzerland, 2022 to 2023

**DOI:** 10.2807/1560-7917.ES.2026.31.18.2500650

**Published:** 2026-05-07

**Authors:** Gaud Catho, Jason Toko, Loïc Fortchantre, Elia Lo Priore, Carlo Balmelli, Lauro Damonti, Philipp Jent, Estelle Moulin, Bruno Grandbastien, Peter W Schreiber, Sarah Tschudin-Sutter, Jan Adam Roth, Lorenzo Ciullini, Claudine Reiber, Walter Zingg, Alexandra U Scherrer, Stephan Harbarth, Niccolò Buetti, on behalf of Swissnoso working group

**Affiliations:** 1 Infection Control Programme and World Health Organization Collaborating Centre, Geneva University Hospitals and Faculty of Medicine, Geneva, Switzerland; 2 Infectious Diseases Division, Central Institute, Valais Hospital, Sion, Switzerland; 3 Division of Infectious Diseases, Department of Medicine, Ente Ospedaliero Cantonale, Lugano, Switzerland; 4 Department of Infectious Diseases, Inselspital, Bern University Hospital, University of Bern, Bern, Switzerland; 5 Infection Prevention and Control Unit, Infectious Diseases Service, Lausanne University Hospital and University of Lausanne, Lausanne, Switzerland; 6 Department of Infectious Diseases and Hospital Epidemiology, University Hospital Zurich and University of Zurich, Zurich, Switzerland; 7 Division of Infectious Diseases and Infection Control, Hospital Epidemiology, University Hospital Basel, University of Basel, Basel, Switzerland; 8 Swissnoso National Center for Infection Control, Bern, Switzerland; 9 Université Paris-Cité, INSERM, IAME, UMR 1137, Paris, France; 10 The members of the Swissono group are acknowledged at the end of the article.

**Keywords:** sensitivity, specificity, healthcare associated infections, CLABSI, bloodstream infection, catheter-infection, Intensive care unit, digital, automated, external validation

## Abstract

**BACKGROUND:**

Accurate, fully automated systems may increase efficiency of healthcare-associated infections (HAI) surveillance.

**AIM:**

We aimed to validate the performance of a fully automated surveillance system for central-line-associated bloodstream infections (CLABSI) in critically ill patients in Switzerland.

**METHODS:**

We conducted a multicentre retrospective study across six secondary and tertiary care hospital networks’ intensive care units (ICU). A centrally hosted, fully automated algorithm was implemented to detect catheter-related bloodstream infections (CRBSI), CLABSI and ICU-onset bloodstream infections (ICU-BSI). Algorithm performance was validated against an anonymised manual review of random samples of positive blood cultures. Incidence data were computed for each hospital.

**RESULTS:**

From January 2022 to December 2023, we analysed 131,166 patient days, 108,719 catheter days and 7,832 positive blood cultures from 1,931 ICU patients. Median age was 65 years (interquartile range (IQR): 53–73), 458 (23.7%) were female. For CLABSI and CRBSI, the algorithm demonstrated a specificity of 95.3% (95% confidence interval (CI): 92.7–97.0), sensitivity of 86.5% (95% CI: 79.8–91.2), positive predictive value of 87.0% (95% CI: 80.4–91.7) and negative predictive value of 95.1% (95% CI: 92.5–96.8). CRBSI/CLABSI and ICU-BSI incidence rates were 3.23/1,000 catheter days (95% CI: 2.91–3.57) and 2.42/1,000 patient days (95% CI: 2.17–2.70), respectively. Most identified microorganisms for CRBSI/CLABSI were *Staphylococcus epidermidis* (15.1%; 53/351), *Enterococcus faecium* (9.1%; 32/351) and *E. faecalis* (5.7%; 20/351).

**CONCLUSIONS:**

We demonstrate feasibility and external validity of a fully automated system for CLABSI surveillance in critically ill patients, supporting its integration into national HAI prevention and control strategies.

**Key public health messages**

**What did you want to address in this study and why?**

We aimed to evaluate the feasibility and performance of a fully automated national surveillance system for central line-associated bloodstream infections (CLABSI) and catheter-related bloodstream infections (CRBSI) in intensive care units. Manual surveillance is resource-intensive and results are variable and delayed, illustrating the need for standardised and timely automated approaches to support infection prevention and control.

**What have we learnt from this study?**

We showed that automated CLABSI surveillance based on electronic health record data is feasible across multiple hospitals with different information systems. The algorithm used to automate the surveillance was successfully applied and validated using data from six hospital networks. The validated algorithm showed high diagnostic accuracy, supporting its reliability for detecting and monitoring CLABSI at national level.

**What are the implications of your findings for public health?**

Automated surveillance offers a sustainable solution to improve the timeliness, comparability and quality of CLABSI surveillance data. National implementation of an automated surveillance system could strengthen infection prevention programmes, enable benchmarking across institutions and inform evidence-based public health strategies to reduce CLABSI incidence and improve patient safety.

## Introduction

Healthcare-associated infections (HAI) cause a large clinical and societal burden in terms of morbidity, mortality and costs [1]. Central-line-associated bloodstream infections (CLABSIs) and catheter-related bloodstream infections (CRBSIs) may increase mortality and hospital length of stay and are largely preventable [2].

The World Health Organization (WHO) considers surveillance one of the essential components of effective prevention of HAI. Surveillance systems provide baseline data, detect increases in incidence, allow comparison over time and between healthcare facilities and enable assessment of the impact of prevention measures [3]. For decades, surveillance of HAI has been based on manual work involving collection of data from health records and classification based on standardised HAI case definitions. Such systems have weaknesses, such as inter-rater variability [4], high workload [5] and delays that limit reactive actions. Over the last decade, there has been progress towards digitalisation of surveillance, reflecting the widespread adoption of electronic health records (EHRs) which provide opportunities to extract some data automatically. Several initiatives emerged, mostly in high-income countries, to transition to automated HAI surveillance [6]. However, few automated surveillance systems have been implemented at national level, and, to our knowledge, none for CLABSI surveillance [7]. We developed and implemented a fully automated surveillance system for CRBSI/CLABSI in critically ill patients, which was first validated internally in one hospital network intensive care unit (ICU) in Switzerland [8].

In this study, we aimed to assess the external validity of a fully automated surveillance system for CRBSI and CLABSI detection in critically ill patients across several hospital networks in Switzerland. As a secondary objective, we aimed to describe incidence trends of CRBSI/CLABSI across participating hospital networks.

## Methods

### Study setting, patients and catheters

This study was conducted in the ICUs of six public secondary- and tertiary-care hospital networks in Switzerland: (i) Ente Ospedaliero Cantonale (EOC) Ticino, (ii) Inselspital (Insel), Bern, (iii) Universitätsspital Basel (USB), Basel, (iv) Universitätsspital Zürich (USZ), Zurich, (v) Centre Hospitalier Universitaires Vaudois (CHUV), Lausanne, and (vi) Hôpitaux Universitaires de Genève (HUG), Geneva (geographic location and hospital size according to patient days of participating hospital networks are shown in Supplementary Figure S1) . The term hospital will be further used in the text and refers to hospital network. All adult patients (≥ 18 years) with at least one ICU stay from 1 January 2022 to 31 December 2023, and with at least one positive blood culture during an ICU stay were included. All short-term central venous catheters (CVCs) in situ during ICU stays were included [9]. All long-term CVCs (e.g*.* Broviac catheter), peripherally inserted central catheters, dialysis catheters and arterial catheters were excluded since long-term CVCs are frequently not accessed during an ICU stay.

### Data sources

At each participating hospital, the following individual patient data were extracted from the clinical data repository by the IT person responsible for the surveillance: patient-level data (age, sex (collected as a binary variable), admission and discharge dates); individual-level CVC data (date of insertion and removal, ward of insertion, insertion site and dwell-time); and microbiological data (blood culture results, other culture results and specimen collection dates). These variables, and their required format, were described in a minimal dataset (MDS) provided to each participating hospital (Supplementary Tables S1–S5 present the data from the MDS). All extracted data were structured and provided in a standardised format. The standardised, securely handled data were then transferred to the coordinating centre at HUG through a dedicated application programming interface (API), where the algorithm was then applied by the IT person in charge of the surveillance at HUG.

### Definitions

Bloodstream infections (BSIs) were classified according to adapted European Centre for Disease Prevention and Control (ECDC) definition criteria, as used in the European point prevalence survey for HAI [10-12]. A BSI episode (CRBSI, CLABSI or ICU-onset BSI (ICU-BSI)) was defined as any positive blood culture with the same pathogen within a time window of 14 days (counted in hours). Only BSI episodes occurring 48 h after ICU admission were considered.

Catheter-related bloodstream infections were defined as a BSI at any time point from the day of CVC insertion up to 48 h after CVC removal, and a blood culture with the same microorganism as a quantitative CVC tip culture of 10^3^ colony-forming units (CFU) per mL or greater [13] (or semiquantitative CVC culture > 15 CFU) [10] (see additional definitions in [8]). Central-line-associated bloodstream infections were defined as a BSI from the day of CVC insertion to 48 h after CVC removal, with the absence of positive culture of other specimens showing the same microorganism within an interval of 72 h before or after the first positive blood culture of the episode. This rule was not applied if the microorganism was classified as a common commensal. The types of other specimens considered were restricted to urine, respiratory tract, bone and joint, abdominal and central nervous system specimens [7]. Intensive care unit-BSI was defined as a BSI when the first positive blood culture of the episode was collected after the patient had spent a minimum of 48 h in the ICU, regardless of the presence of a CVC.

A common commensal was considered as a true pathogen when the same common commensal was present in at least two positive blood cultures within 48 h, otherwise it was classified as a contamination. The list of common commensals was based on the United States (US) Centers for Disease Control and Prevention (CDC) National Healthcare Safety Network (NHSN) Organism Category classification used for the surveillance of CLABSI [14].

To limit complexity, polymicrobial blood cultures were considered separate episodes. A set of blood cultures (two vials) was counted as a single positive blood culture. Catheter days and patient days were counted in hours and provided by each hospital as monthly aggregated data. When missing, catheter insertion and removal dates were imputed with ICU admission and discharge dates, respectively. Key differences between definitions used and ECDC definitions are described in the Supplementary material.

### Development of the fully automated algorithm

The fully automated algorithm for CRBSI, CLABSI and ICU-BSI detection (Supplementary Figure S2 represents the fully automated algorithm) was developed and internally validated at HUG, as previously reported [8]. In brief, the following rules were applied sequentially to all positive blood cultures from the study population: (i) grouping positive blood cultures by patient, hospital stay and microorganism, (ii) classifying common commensals as either contaminants or true pathogens, (iii) grouping positive blood cultures with the same pathogen into BSI episodes, (iv) assessing the presence or absence of a CVC, (v) determining the presence of positive cultures with the same microorganism as in the blood culture from other specimen types, and (vi) checking for a CVC tip culture positive for the same microorganism above the defined threshold.

### Outcomes

Catheter-related bloodstream infection and CLABSI (CRBSI/CLABSI) were combined as the primary outcome, since they are different definitions used for central-line BSIs and both clinically relevant [15]. Intensive care unit-BSI was the secondary outcome.

### Validation of the automated algorithm

The automated algorithm was externally validated against the reference standard, defined here as the manual classification of positive blood cultures. For validation, we randomly selected positive blood cultures from the study population (adult patients with at least one positive blood culture during ICU stay). We based our validation sample size on two different samples: a random sample of positive blood cultures was selected independently of their classification by the fully automated surveillance system (Random sample 1) and a second random sample consisting of positive blood cultures classified as CRBSI or CLABSI by the automated algorithm (Random sample 2; Supplementary Figure S2 represents the fully automated algorithm with the two random samples). Blood cultures from both random samples were manually classified at each hospital by one or two independent infectious diseases specialists (or specialised study nurses) who had no knowledge about the automated algorithm’s classification. Manual classification was performed using the same definitions as those applied by the automated algorithm (as described above). Each positive blood culture was classified into one of four categories: (i) contamination, (ii) BSI not attributable to ICU, (iii) ICU-BSI, (iv) CLABSI or CRBSI. Classification was performed at the episode level rather than distinguishing individual cultures as original vs copy strains. After the initial evaluation, the automated algorithm's classification was revealed and the results were compared.

### Sample size calculation

Based on the best available dataset (blood culture data at HUG in 2023, n = 1,487) with a type 1 error of 5%, an expected sensitivity of 90%, a prevalence of 10% of CLABSI and CRBSI in all positive blood cultures and a marginal error of 10%, we estimated that 407 positive blood cultures needed to be included in Random sample 1 [16]. According to the total number of CRBSI/CLABSI episodes predicted using the automated algorithm based on blood culture data at HUG in 2023 (n = 63) with a margin of error of 5%, a confidence level of 95% and a likely proportion of 75%, we estimated that 177 CRBSI/CLABSI episodes would be needed in Random sample 2 [17]. The total (Random samples 1 and 2) estimated sample size was 584.

### Statistical analysis

First, using our validation samples, we computed specificity, sensitivity, positive and negative predictive values of the automated algorithm for the primary outcome (CRBSI/CLABSI), the secondary outcome (ICU-BSI), and both outcomes combined (CRBSI/CLABSI and ICU-BSI) compared with manual classification defined as the reference standard [18]. Confidence intervals (CI) for specificity, sensitivity, positive and negative predictive values were performed using the Wilson score interval method [19]. The statistical unit for this analysis was positive blood cultures.

Second, using the whole population from January 2022 to December 2023, we compared the characteristics of the different type of episodes of CLABSI/CRBSI and ICU-BSI across all hospital networks (age, sex, delay from ICU admission, microorganism distribution) using chi-square (or Fisher’s exact) or Wilcoxon tests for categorical and numeric variables, respectively.

Third, we computed incidence rates for CRBSI, CLABSI and ICU-BSI, stratified by hospital network and month and across all hospital networks for the study period using the automated algorithm. Incidence rates were calculated using catheter days as denominator for CRBSI and CLABSI and patient days for ICU-BSI. Finally, we presented trends of each type of event by hospital and by month across the study period. All calculations were performed using R software version 4.1.3 [20].

## Results

**Figure 1:**
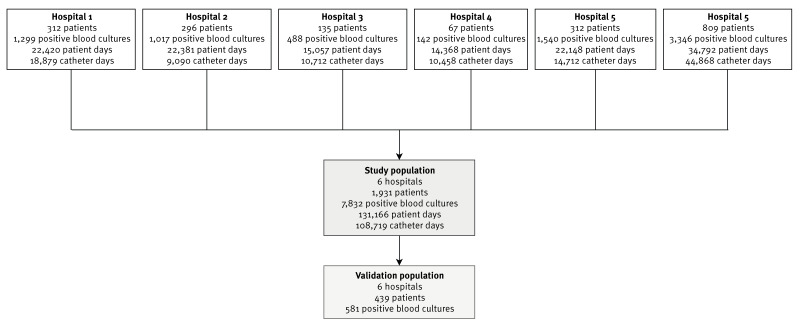
Study flowchart

From 1 January 2022 to 31 December 2023, a total of 131,166 patient days, 108,719 catheter days and 7,832 positive blood cultures from 1,931 critically ill patients were collected (Figure 1).

The median age of the patients was 65 years (interquartile range (IQR): 53–73), 458 (23.7%) were female and 1,473 (76.3%) were males.

### Validation of the automated algorithm

**Figure 2:**
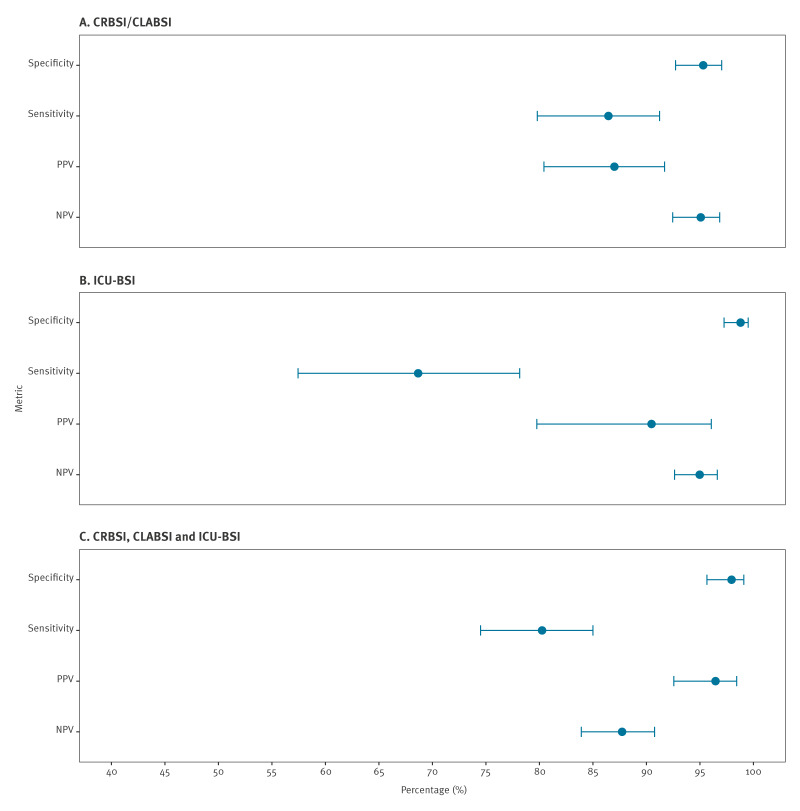
Validation metrics for catheter-related bloodstream infection, central-line-associated bloodstream infection and intensive care unit-onset bloodstream infection automated surveillance, Switzerland

BSI: bloodstream infection; CLABSI: central-line associated bloodstream infection; CRBSI: catheter-related bloodstream infection; ICU: intensive care unit; NPV: negative predictive value; PPV: positive predictive value.

From this study population, 581 blood cultures were randomly selected for validation (407 in Random sample 1 and 174 in Random sample 2). Figure 2 shows the overall performance of the automated algorithm for each outcome.

For the validation, 581 blood cultures were randomly selected from all hospital networks. The x-axis represents different percentages with 95% confidence intervals.

For the primary outcome (CRBSI/CLABSI), the algorithm showed a specificity of 95.3% (95% CI: 92.7–97.0), a sensitivity of 86.5% (95% CI: 79.8–91.2), a positive predictive value of 87.0% (95% CI: 80.4–91.7) and a negative predictive value of 95.1% (95% CI: 92.5–96.8) (Supplementary Table S6 shows a contingency table and the performance metrics for the primary outcome). For ICU-BSI, the algorithm showed a specificity of 98.8% (95% CI: 97.2–99.5), sensitivity of 68.7% (95% CI: 57.4–78.2), a positive predictive value of 90.5% (95% CI: 80.4–91.7) and a negative predictive value of 94.9% (95% CI: 92.5–96.8) (Supplementary Tables S7–S8 show contingency tables and performance metrics for the secondary outcome). Performance metrics by hospital network are provided in Supplementary Table S9).

### Description of the type of episodes

From 1 January 2022 to 31 December 2023, 12 CRBSI, 339 CLABSI and 318 ICU-BSI episodes were identified by the automated algorithm in the six hospital networks. Characteristics of the episodes are described in Table 1. Hospitals 1 and 6 accounted for 55.6% (195/351) of the total number of CRBSI/CLABSI episodes and 62.2% (198/318) of the total number of ICU-BSI episodes.

**Table 1:** Description of catheter-related bloodstream infections, central-line-associated bloodstream infections and intensive care unit-onset bloodstream infections, Switzerland, January 2022–December 2023 (n = 669 bloodstream infection episodes)

Characteristics	CRBSI/CLABSI n = 351		ICU onset BSI n = 318		p value
	n	%	n	%	
Female	111	31.6	92	28.9	0.501
Age in years, median (IQR)	62 (50–72)		64 (51–72)		0.602
Delay from ICU admission in days, median, (IQR)	9 (5–16.5)		9 (6–20)		0.3679
**Type of microorganisms**					
Common commensal	85	24.2	34	10.7	< 0.001
True pathogen	266	75.8	284	89.3	NA
**Most frequently observed microorganisms**					
*Candida albicans*	16	4.5	24	7.5	NA
*Candida glabrata*	13	3.7	8	2.5	
*Enterococcus faecalis*	20	5.7	22	6.9	
*Enterococcus faecium*	32	9.1	30	9.4	
*Escherichia coli*	16	4.5	26	8.2	
*Klebsiella pneumoniae*	18	5.1	30	9.4	
*Pseudomonas aeruginosa*	7	2.3	20	6.3	
*Serratia marcescens*	8	2.3	24	7.5	
*Staphylococcus aureus*	18	5.1	29	9.1	
*Staphylococcus epidermidis*	53	15.1	26	8.2	

CLABSI: central-line associated bloodstream infection; CRBSI: catheter-related bloodstream infection; ICU: intensive care unit; ICU-BSI: ICU onset bloodstream infection; IQR: interquartile range; NA: not applicable.

The most frequently isolated microorganisms for CRBSI/CLABSI episodes were *Staphylococcus epidermidis* (15.1%) *Enterococcus faecium* (9.1%) and *E. faecalis* (5.7%) (Table 1).

### Incidence of intensive care unit-onset bloodstream infections, central-line-associated bloodstream infections and catheter-related bloodstream infections

The overall incidence rate of CRBSI and CLABSI combined was 3.23 per 1,000 catheter days (95% CI: 2.91–3.57) and the overall incidence rate of ICU-BSI per 1,000 patient days was 2.42 per 1,000 patient days (95% CI: 2.17–2.70) (Table 2).

**Table 2:** Number of events and incidence rates by hospital, Switzerland, January 2022–December 2023, (n = 351 catheter-related bloodstream infection and central-line-associated bloodstream infection episodes)

Characteristics	Hospital 1		Hospital 2		Hospital 3		Hospital 4		Hospital 5		Hospital 6		All hospitals	
**Number of events**														
**Patient days**	22,420		22,381		15,057		14,368		22,148		34,792		131,166	
**Catheter days**	18,879		9,090		10,712		10,458		14,712		44,868		108,719	
**CRBSI and CLABSI episodes**	80		57		29		15		55		115		351	
**CRBSI episodes**	3		5		0		4		0		0		12	
**CLABSI episodes**	77		52		29		11		55		115		339	
**ICU-BSI episodes**	60		57		25		3		35		138		318	
**Incidence rates**														
**Incidence of**	IR	95% CI	IR	95% CI	IR	95% CI	IR	95% CI	IR	95% CI	IR	95% CI	IR	95% CI
**CRBSI and CLABSI per 1,000 catheter days**	4.24	3.34–5.19	6.27	4.73–7.92	2.71	1.77–3.73	1.43	0.76–2.20	3.74	2.79–4.76	2.56	2.10–3.03	3.23	2.91–3.57
**ICU-BSI per 1,000 patient days**	2.68	2.01–3.39	2.55	1.92–3.22	1.66	1.06–2.32	0.21	0.00–0.49	1.58	1.08–2.12	3.97	3.31–4.63	2.42	2.17–2.70
**CRBSI per 1,000 catheter days**	0.16	0.00–0.37	0.55	0.11–1.10	0	0.00–0.00	0.38	0.10–0.76	0	0.00–0.00	0	0.00–0.00	0.11	0.06–0.17
**CLABSI per 1,000 catheter days**	4.08	3.18–5.03	5.72	4.18–7.37	2.71	1.77–3.73	1.05	0.48–1.72	3.74	2.79–4.76	2.56	2.10–3.03	3.12	2.79–3.46

BSI: bloodstream infection; CI: confidence interval; CLABSI: central-line associated bloodstream infection; CRBSI: catheter-related bloodstream infection; ICU: intensive care unit; IR: incidence rate.

**Figure 3:**
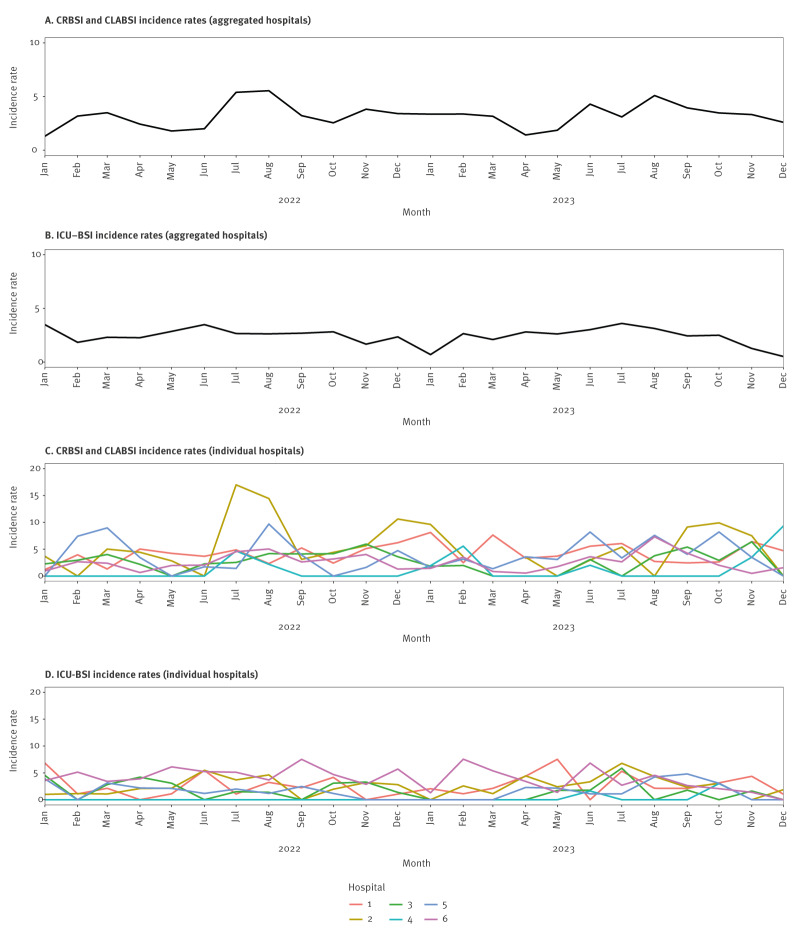
Time trends aggregated for participating hospital networks and stratified individual hospitals, Switzerland, January 2022–December 2023

BSI: bloodstream infection; CLABSI: central-line associated bloodstream infection; CRBSI: catheter-related bloodstream infection; ICU: intensive care unit.

Incidence rates of CLABSI/CRBSI ranged from 1.43 (Hospital 4) to 6.27 (Hospital 2) per 1,000 catheter days. Incidence rates for aggregated hospitals and by hospital, type of event (CRBSI, CLABSI or ICU-BSI) and month are presented in Figure 3.

For CRBSI and CLABSI, incidence rates are calculated per 1,000 catheter days. For ICU-BSI, incidence rates are calculated per 1,000 patient days.

## Discussion

To our knowledge, this study represents one of the first national deployment and external validation of a fully automated surveillance system for CLABSI and CRBSI in ICUs. Our automated system demonstrated robust diagnostic accuracy metrics, with a specificity of 95.3% and sensitivity of 86.5%, supporting its potential as a reliable tool for real-time monitoring of this type of HAI. This study also highlights the feasibility of deploying a centralised, automated surveillance system for CLABSI at national level. Each participating hospital successfully extracted, standardised and achieved secure transmission of data.

A review of automated surveillance systems showed them to be heterogeneous in terms of definitions, datasets and denominators used, and overall demonstrated encouraging performance, with pooled specificity and sensitivity of 0.86 (95% CI: 0.79–0.92) and 0.88 (95% CI: 0.84–0.91), respectively [21]. Parameters for our automated surveillance system were selected based on a systematic review and meta-regression of existing automated systems, identifying those associated with optimal specificity and sensitivity [22]. Central-line-associated bloodstream infections remain a key focus of infection prevention programmes due to their high morbidity, mortality and preventability. Compared with hospital-onset BSI surveillance, CLABSI surveillance provides a readily interpretable and actionable indicator that can directly inform targeted infection control interventions. Reliable surveillance is critical to guide interventions, but current manual data collection strategies are resource-intensive, inconsistent and slow to deliver actionable data. Our study addresses these limitations by validating an automated system that allows standardised CLABSI detection in diverse institutions, with the potential for real-time implementation.

When comparing our results with existing data, the performance of the automated system is in line with prior studies focusing on algorithm-based surveillance of HAIs and specifically of CLABSI [22]. The observed incidence rate of 3.23 per 1,000 catheter days for CRBSI and CLABSI aligns with previously reported figures in high-income countries and underscores the clinical relevance of the detected events. In 2021, ECDC reported an aggregated CLABSI incidence rate in ICUs of 4.1 per 1,000 catheter days and an aggregated ICU-BSI incidence rate of 6.9 per 1,000 patient days across eight European countries [23]. The reported incidence rate of CLABSI in the US by the CDC NSHN surveillance network was 0.84 per 1,000 catheter days (2015–2017) [24]. In a large prospective cohort study conducted from 2004 to 2022 across 181 ICUs in Asian countries (also based on CDC NHSN criteria) including 853,604 patient days, the reported pooled CLABSI rate was 5.08 per 1,000 catheter days [25].

Importantly, comparisons between incidence rates from automated and manual surveillance should be interpreted with caution, given differences of case definitions. Our algorithm, for example, attributes BSIs to a catheter source only when the same organism is not identified in other microbiological samples at the same time. Moreover, it is conceivable that secondary sources of infections are more frequently detected using a manual review of patients’ charts, since automated systems miss incorrectly labelled specimens, thus lowering CLABSI/CRBSI incidence rates. We deliberately excluded low-specificity samples, such as superficial swabs, which may have led to misclassification and underestimation of CLABSI rates. Conversely, primary infection sites may not always yield positive cultures, potentially resulting in overestimation of CLABSI rates. These choices reflect a trade-off between specificity and sensitivity, in a way that makes the surveillance system most useful for fair and meaningful comparisons between hospitals or time periods. Our aim was not to replicate manual adjudication, but to establish a standardised, automated system for large-scale performance monitoring.

Historically, HAI surveillance has relied on manual processes involving retrospective data collection and expert case adjudication using predefined criteria. While these methods remain the reference standard, they are labour-intensive, prone to inter-rater variability and deliver results with delays that hinder timely intervention [5,6,26-28]. The growing availability of structured EHR data allows the transition from manual to automated systems for surveillance of HAI which offer better standardisation, reduce workload and provide more timely results [29]. However, the transition is slow, is limited to research settings, and no national automated surveillance system for CLABSI has yet been implemented [30].

In the field of automated surveillance, we can distinguish fully and semi-automated approaches. Fully automated systems apply predefined rules to structured data, eliminating the need for expert classification, unlike semi-automated systems that still require manual review of flagged cases. Our fully automated surveillance system is based only on structured routine data from local databases. Using a stepwise logic sequence, it classifies positive blood cultures into contamination, non-ICU BSI onset, ICU-BSI, CLABSI and CRBSI categories. While artificial intelligence and more complex models are gaining attention [31,32] and may provide additional help in automating HAI surveillance in the future (for example, the use of natural language processing to extract information from unstructured medical notes or complex models to identify the source of a BSI), our choice of a simple rule-based approach facilitates broad implementation and reduces technical barriers and computing resources for hospitals. Despite varying IT infrastructures, all participating institutions—whether using in-house or commercial EHR systems—were able to extract the necessary data. The median time was 104 IT hours per hospital to set up the surveillance system (data not shown). We consider this an acceptable figure to allow broad implementation of the system, even in countries other than Switzerland with a similar EHR infrastructure.

Automated surveillance for CLABSI improves efficiency and allows continuous monitoring, but several limitations exist. Its accuracy relies on the completeness and correctness of EHR data, including microbiology results, catheter insertion dates and clinical documentation. Automated algorithms may misclassify BSIs, particularly in distinguishing true CLABSI from contamination or secondary bacteraemia. Clinically suspected CLABSI without positive cultures are missed. Accurate attribution of infection to a specific central vascular catheter and timing relative to line insertion also remains challenging. In addition, surveillance rules and data structures require regular validation and updates to reflect changes in definitions, coding practices or local workflows which can substantially affect performance.

Future efforts should focus on expanding this system to all Swiss hospitals to allow nationwide benchmarking of CLABSI rates. Currently supported by the Swiss Federal Office of Public Health, this centrally coordinated surveillance initiative will invite participation from all Swiss hospitals. As seen in the pilot phase, participating hospitals will transmit pseudonymised data to a coordinating centre, which will generate national incidence rates and periodically provide feedback reports with graphical summaries to participating hospitals. Public health authorities, hospital quality managers and infection prevention teams could use the data for public health purposes, patient safety initiatives and prevention strategies. This could enable trend analysis and inform resource allocation for HAI prevention strategies. Involving infection prevention teams and clinicians in interpreting the surveillance outputs will remain crucial. In addition, the system could be adapted for use outside the ICU, provided that the relevant catheter data are available.

Despite the promising results, several challenges must be mentioned. First, surveillance was limited to ICUs, although CLABSI also occurs in other hospital settings. However, the system is readily adaptable to other medical specialties if data are electronically available. Second, external validation was conducted retrospectively on a blood culture level; prospective validation will be needed to confirm performance in real-time settings. Third, although the algorithm was compared against manual adjudication using consistent case definitions, these definitions were adapted for automation and may differ from those used in conventional manual surveillance systems (e.g. ECDC definitions). Fourth, we observed a lower sensitivity for ICU-BSI data, compared with CLABSI/CRBSI data, our primary outcome. This discrepancy is due to episodes fulfilling the criteria for ICU-BSI but classified by the algorithm as CLABSI/CRBSI being counted as false negatives. This methodological choice artificially lowered the observed sensitivity of ICU-BSI. Finally, achieving data harmonisation across institutions required considerable effort, particularly for microbiology and catheter datasets. Successful implementation depended on strong collaboration between IT personnel and clinical teams.

## Conclusions

Despite its recognised role in infection prevention, national programmes for largely preventable HAI such as CLABSI are still lacking in many countries. We demonstrated the possibility to apply fully automated surveillance of CLABSI across several hospital networks in Switzerland. Our findings provide the basis for implementing automated systems that can reduce feedback delays, promote standardisation and improve comparability across institutions. Automated CLABSI surveillance systems can serve as powerful tools to drive preventive efforts at all levels. Moreover, our experience may serve as a model for other countries aiming to modernise and expand their HAI surveillance infrastructure.

## Data Availability

The datasets used and/or analysed during the current study are available from the corresponding author on reasonable request.

## Supplementary Data

487000 Supplement
