# Supplementary material for: First results of a national deployment of a fully automated central-line-associated bloodstream infection (CLABSI) surveillance system, Switzerland, 2022 to 2023
Source: Euro Surveill. 2026 May 7;31(18):2500650. doi: 10.2807/1560-7917.ES.2026.31.18.2500650 (PMC13170418; doi:10.2807/1560-7917.ES.2026.31.18.2500650)

This supplementary material is hosted by *Eurosurveillance* as supporting information alongside the article 'First results of a national deployment of a fully automated central-line-associated bloodstream infection (CLABSI) surveillance system, Switzerland, 2022 to 2023', on behalf of the authors, who remain responsible for the accuracy and appropriateness of the content. The same standards for ethics, copyright, attributions and permissions as for the article apply. Supplements are not edited by *Eurosurveillance* and the journal is not responsible for the maintenance of any links or email addresses provided therein.

Additional Definitions

The following criteria (used in ECDC definition for CRBSI) were not included in the automated algorithm for CRBSI: *(a)* quantitative blood culture ratio CVC blood sample/peripheral blood sample >5; *(b)* differential time of blood culture positivity (DTP); *(c)* positive culture with the same microorganism from pus at the insertion site and *(d)* clinical criterion of improvement within 48 hours of catheter removal. The criteria (a) and (b) were not implemented in the automated algorithm because they were not systematically provided by microbiology labs. The criteria (c) and (d) were not implemented due to the difficulty of automatically extracting and correctly identifying the data.

Supplementary Tables S1-S5: Minimal dataset (MDS)

The MDS included five tables including mandatories and optional variables.
We developed patients, encounters, catheters, cultures, denominators tables as described below. All variables were structured according to predefined definitions.

Supplementary Table S1: Patients

Variable Name	Description	Mandatory	Type	Possible Values	Format
patient_pseudo_id	Pseudonymized patient ID	Yes	string		
gender	Gender as in EHR	Yes	string	M = male<br>F = female<br>O = Other<br>U =Unknown	
birth_year	Year of birth	Yes	numeric		YYYY
death_date	Date of in-hospital death	No	date		YYYY/MM/DD
in_hospital_mortality	Died during hospital stay	No	boolean	YES / NO	
data_provider_institution	Data source institution	Yes	string		

Legend: ID = Identifier; EHR = Electronic Health Record

Supplementary Table S2: Encounters

Variable Name	Description	Mandatory	Type	Format
patient_pseudo_id	Pseudonymized patient ID	Yes	string	

hospital_encounter_id	Hospital encounter ID	Yes	string	
hospital_encounter_begin_date	Hospital admission date	No	Timestamp UTC ISO	YYYY-MM-DD'T'HH:mm:ss'Z'
hospital_encounter_dicharge_date	Hospital discharge date	No	Timestamp UTC ISO	YYYY-MM-DD'T'HH:mm:ss'Z'
begin_date_icu	ICU admission date	Yes	Timestamp UTC ISO	YYYY-MM-DD'T'HH:mm:ss'Z'
discharge_date_icu	ICU discharge date	Yes	Timestamp UTC ISO	YYYY-MM-DD'T'HH:mm:ss'Z'
data_provider_institution	Data source institution	Yes	string	

Legend: ID = Identifier; ICU = Intensive Care Unit; UTC = Coordinated Universal Time; ISO = International Organization for Standardization

Supplementary Table S3: Cultures

Variable Name	Description	Mandatory	Type	Possible Values	Format
patient_pseudo_id	Pseudonymized patient ID	Yes	string		
hospital_encounter_id	Hospital encounter identifier	Yes	string		
lab_id	Unique lab sample identifier	Yes	string		
lab_sample_date	Sample collection date	Yes	Timestamp UTC ISO		YYYY-MM-DD'T'HH:mm:ss'Z'
lab_specimen_local_code	Local code for sample type	Yes	numeric		
lab_specimen_local_label	Local label of the specimen	Yes	string		
lab_specimen_category	Specimen category	Yes	string	blood; catheter_tip; respiratory_tract; abdominal; central_nervous_system; urinary; bone_joint	
lab_specimen_SNOMED	Specimen type (SNOMED CT)	Yes	string		
microorg_local_ID	Local ID of microorganism	Yes	numeric		

microorg_local_label	Local label of microorganism	Yes	string		
microorg_NHSN_label	NHSN microorganism label	Yes	string		
microorg_SNOMED_code	SNOMED code of microorganism	Yes	numeric		
lab_cc_value	Common contaminant (NHSN list)	Yes	boolean	YES / NO	
lab_unit	Unit where sample was taken	Yes	string		
unit_ecdc_classification	ECDC unit classification	Yes	string	ICUMED; ICUSUR; ICUSPEC; ICUMIX; ICUOTH; ICUCOV	
microorg_tip_quantification	Pathogen count (CFU/mL, catheter tip only)	Yes	numeric		
microorg_tip_semi_quantification	Pathogen category (CFU/mL, catheter tip only)	Yes	string		
microorg_tip_semi_quanti_pos	Categorical quantification positive?	Yes	boolean	TRUE / FALSE	
data_provider_institution	Data source institution	Yes	string	CHUV; HUG; USZ; USB; EOC; Insel	

Legend: ID = Identifier; SNOMED = Systematized Nomenclature of Medicine; NHSN = National Healthcare Safety Network; ECDC = European Centre for Disease Prevention and Control; ICU = Intensive Care Unit

Supplementary Table S4: Catheters

**Variable Name**	**Description**	**Mandatory**	**Type**	**Possible Values**	**Format**
patient_pseudo_id	Pseudonymized patient ID	Yes	string		
hospital_encounter_id	Hospital encounter identifier	Yes	string		
catheter_id	Catheter unique identifier	Yes	string		

catheter_type_local	Local label of catheter type	Yes	string		
catheter_type_SNOMED	SNOMED CT label for catheter type	Yes	string		
catheter_insertion_date	Date of catheter insertion	Yes	Timestamp UTC ISO		YYYY-MM-DD'T'HH:mm:ss'Z'
catheter_removal_date	Date of catheter removal	Yes	Timestamp UTC ISO		YYYY-MM-DD'T'HH:mm:ss'Z'
catheter_insertion_site_local	Local label of insertion site	No	string		
catheter_insertion_site_category	Standardised site of insertion	No	string		
catheter_insertion_care_unit	Unit where catheter was inserted	Yes	string	ICUMED; ICUSUR; ICUSPEC; ICUMIX; ICUOTH; ICUCOV	
catheter_insertion_care_unit_ecdc	ECDC classification of insertion unit	No	string		
data_provider_institution	Institution providing the data	Yes	string	CHUV; HUG; USZ; USB; EOC; Insel	

Legend: ID = Identifier; SNOMED = Systematized Nomenclature of Medicine – Clinical Terms; ECDC = European Centre for Disease Prevention and Control; ICU = Intensive Care Unit; UTC = Coordinated Universal Time; ISO = International Organization for Standardization

Supplementary Table S5: Denominators

Variable Name	Description	Mandatory	Type	Format	Possible Values
month_year	Year and month of the extraction	Yes	string	MM-YYYY	
patient_days	Number of patient days (counted in hours, 1 day = 24h)	Yes	numeric		
catheter_days	Number of catheter days (counted in hours, 1 day = 24h)	Yes	numeric		
unit_name	Local name of the unit	Yes	string		

unit_ecdc_classification	Classification of the unit (ECDC)	No	string		ICUMED; ICUSUR; ICUSPEC; ICUMIX; ICUOTH; ICUCOV
data_provider_institution	Institution from which the data are coming	Yes	string		CHUV; HUG; USZ; USB; EOC; Insel

Legend: ECDC = European Centre for Disease Prevention and Control; ICU = Intensive Care Unit

Supplementary Table S6: Validation metrics for CRBSI/CLABSI

		Manual surveillance	
		**CLABSI/CRBSI**	**No CLABSI/CRBSI**
**Automated surveillance**	**CLABSI/CRBSI**	134 (TP)	21 (FN)
	**No CLABSI/CRBSI**	20 (FP)	406 (TN)
	**value**	**Lower IC95%**	**Upper IC95%**
**Sensitivity**	86.45	79.81	91.23
**Specificity**	95.31	92.72	97.03
**PPV**	87.01	80.42	91.7
**NPV**	95.08	92.46	96.85

TP: true positive, FP: false positive; FN: false negative; TN: true negative; PPV: positive predictive value.

Supplementary Table S7: Validation metrics for ICU-onset BSI

		Manual surveillance	
		**ICU-BSI**	**No ICU-BSI**
**Automated surveillance**	**ICU-BSI**	57 (TP)	26 (FN)
	**No ICU-BSI**	6 (FP)	492 (TN)
	**value**	**Lower IC95%**	**Upper IC95%**
**Sensitivity**	68.67	57.44	78.16
**Specificity**	98.8	97.26	99.51
**PPV**	90.48	80.42	91.7
**NPV**	94.98	92.46	96.85

Supplementary Table S8: Validation metrics for CRBSI, CLABSI and ICU-BSI

		Manual surveillance	
		191 (TP)	47 (FN)

**Automated surveillance**		7 (FP)	336 (TN)
	**value**	**Lower IC95%**	**Upper IC95%**
**Sensitivity**	80.25	74.5	85
**Specificity**	97.96	95.66	99.1
**PPV**	96.46	92.56	98.44
**NPV**	87.73	83.92	90.76

Supplementary Table S9. Performance metrics by institution for CLABSI/CRBSI

**Hospital network**	**Metric**	**Value**	**Lower IC95%**	**Upper IC95%**
Hosp 1	Sensitivity	89.29	70.63	97.19
	Specificity	83.87	71.87	91.59
	PPV	71.43	53.48	84.76
	NPV	94.55	83.93	98.58
Hosp 2	Sensitivity	100	86.27	100
	Specificity	98.33	89.86	99.91
	PPV	96.88	82	99.84
	NPV	100	92.38	100
Hosp 3	Sensitivity	78.38	61.34	89.58
	Specificity	100	92.84	100
	PPV	100	85.44	100
	NPV	88.73	78.47	94.66
Hosp 4	Sensitivity	90.91	69.38	98.41
	Specificity	98.72	92.09	99.93
	PPV	95.24	74.13	99.75
	NPV	97.47	90.31	99.56
Hosp 5	Sensitivity	100	67.86	100
	Specificity	94.38	86.78	97.91
	PPV	68.75	41.48	87.87
	NPV	100	94.55	100
Hosp 6	Sensitivity	69.23	48.1	84.91
	Specificity	95.95	87.82	98.95

	PPV	85.71	62.64	96.24
	NPV	89.87	80.5	95.21

Supplementary Figure S1

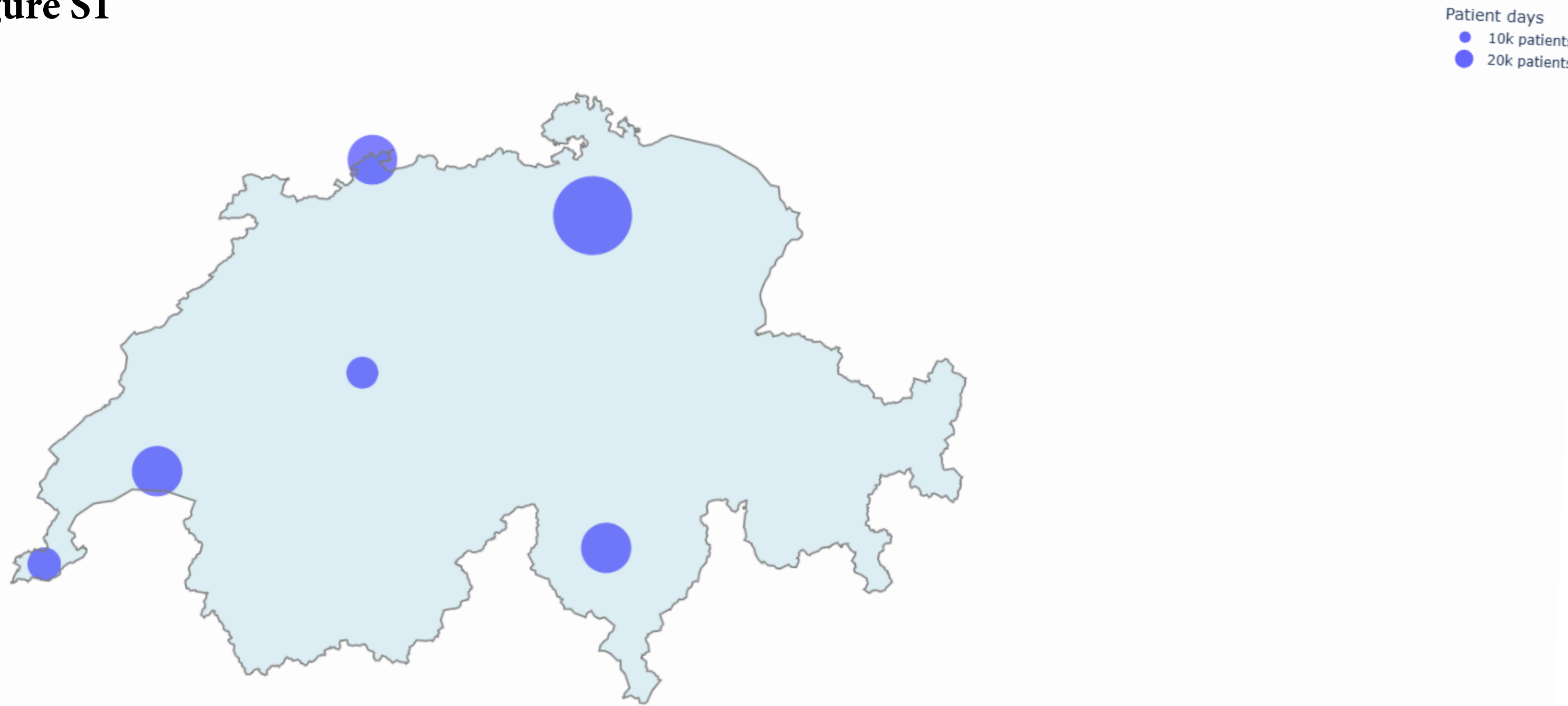

Supplementary Figure S2

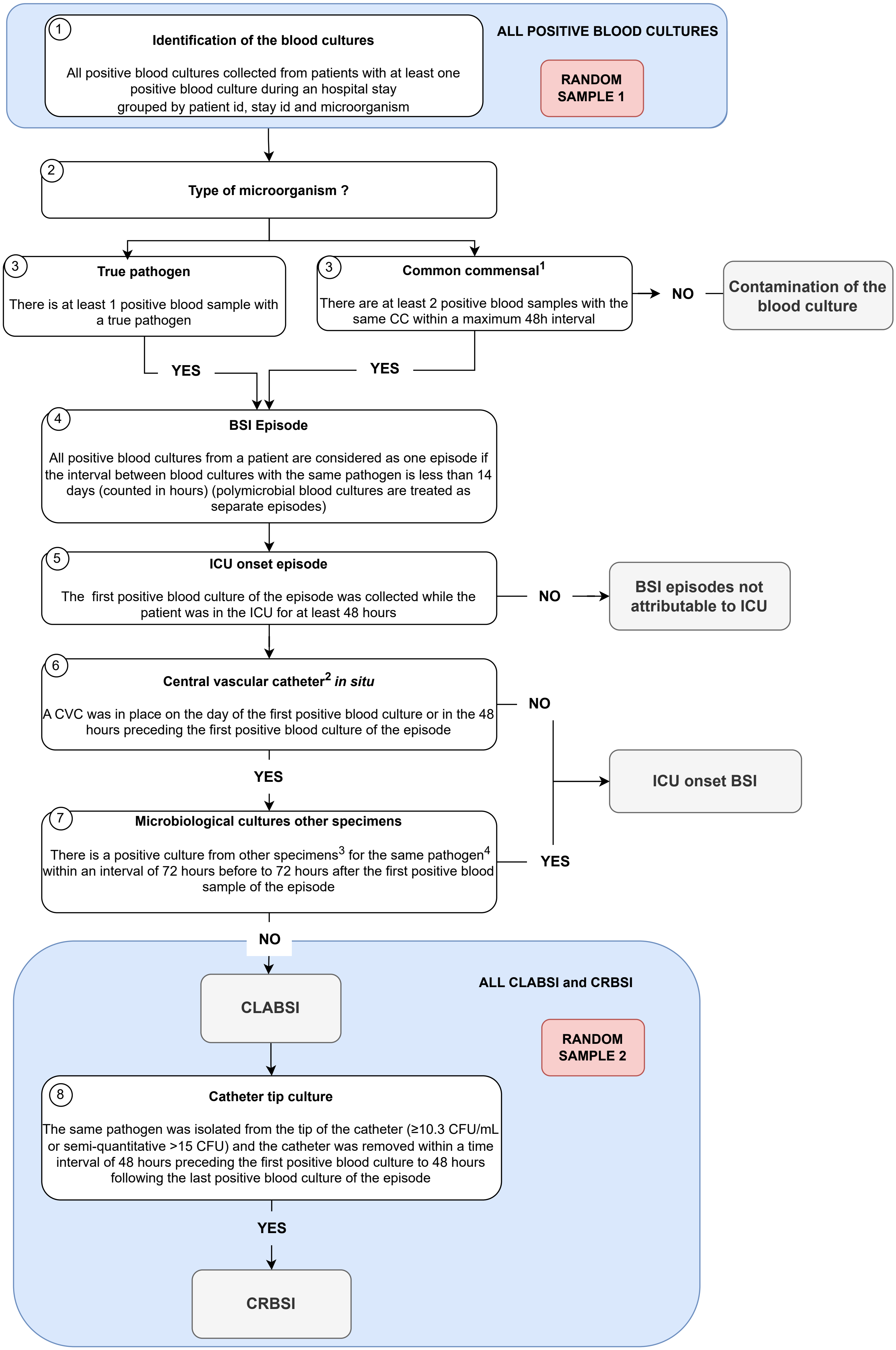

Supplement: Supplement [file 2500650_Supplement.pdf]
